# Skeletal and Dentoalveolar Changes Following the Functional Treatment of Skeletal Class II Growing Patients: A Systematic Review and Meta-Analysis

**DOI:** 10.7759/cureus.109730

**Published:** 2026-05-27

**Authors:** Dima M. Almrayati, Mohammad Y. Hajeer, Mohammad Khursheed Alam, Ahmad S. Burhan, Mowaffak A. Ajaj, Huda Abutayyem, Samer T. Jaber

**Affiliations:** 1 Department of Orthodontics, Faculty of Dentistry, University of Damascus, Damascus, SYR; 2 Department of Preventive Dental Science, College of Dentistry, Jouf University, Sakaka, SAU; 3 Department of Clinical Sciences, Center of Medical and Bio-Allied Health Sciences Research, College of Dentistry, Ajman University, Ajman, ARE; 4 Department of Orthodontics, Faculty of Dentistry, Al-Watanyia Private University, Hama, SYR

**Keywords:** activator, bionator, cephalometric assessment, class ii malocclusion, dentoalveolar change, functional treatment, growing patients, skeletal change, skeletal class ii malocclusion, twin-block

## Abstract

This systematic review was conducted to critically assess the effects of removable and fixed functional appliances (FFAs) on skeletal and dentoalveolar outcomes in growing patients with skeletal Class II malocclusion. In September 2025, a comprehensive literature search was performed across eight electronic databases. Randomized controlled trials (RCTs) and controlled clinical trials (CCTs) evaluating skeletal and dentoalveolar changes after treatment with removable functional appliances (RFAs) or FFAs were considered eligible. The included studies involved patients aged 9-14 years who underwent treatment with either RFAs or FFAs. Treatment outcomes were evaluated using cephalometric radiographic measurements. Study selection, data extraction, and risk-of-bias evaluation were independently conducted using the Cochrane risk-of-bias tools, while the certainty of the evidence was assessed according to the Grading of Recommendations Assessment, Development, and Evaluation (GRADE) methodology. A total of six studies, including four RCTs and two CCTs, comprising 276 patients, fulfilled the eligibility criteria and were included in the final analysis. Overall, both RFAs and FFAs were effective in improving skeletal Class II malocclusion following active treatment, with correction resulting from combined skeletal and dentoalveolar changes. Most appliances, including Activator, Twin-block, and Aesthetic Twin-block (ATB), produced favorable sagittal changes, primarily mediated by mandibular advancement and modest restriction of maxillary growth, in conjunction with dentoalveolar adaptations. In contrast, the Trainer (T4K®) exhibited limited skeletal effects, suggesting that its mechanism of action is predominantly dentoalveolar. With regard to vertical changes, no clinically significant effects were observed for the Activator, Twin-block, ATB, or Trainer (T4K®), indicating minimal impact on lower facial height during the course of treatment. Conversely, the Bite-Jumping Appliance (BJA) was associated with increased facial height. The overall certainty of the evidence ranged from low to moderate, primarily due to methodological limitations and inter-study heterogeneity. In conclusion, both RFAs and FFAs - excluding the Trainer (T4K®) - are effective in the correction of skeletal Class II malocclusion in growing patients, primarily through maxillary growth restriction, increased mandibular length, and favorable dentoalveolar adaptations. No clinically significant short-term vertical changes were observed for most appliances; however, the certainty of this finding remains limited. Further well-designed, long-term RCTs directly comparing these treatment modalities are required to strengthen the evidence base and inform clinical decision-making. This systematic review was prospectively registered in the PROSPERO database (ID: CRD42025633113).

## Introduction and background

Class II malocclusion is among the most commonly observed orthodontic conditions in clinical practice. It occurs with a global frequency of 20%, and 22.9% of Caucasians are affected [1]. According to Angle's classification, Class II malocclusion is further categorized into Division 1 and Division 2. This malocclusion is caused by a combination of hereditary, functional, and environmental factors [2]. Class II malocclusions can be treated using one of three modalities: growth modification, camouflage (with or without extraction), or orthognathic surgery [3]. As a result, the most appropriate treatment plan is selected after considering the etiology, the severity of the structural abnormality, and the patient's age [4].

Functional therapy is considered to be the best therapeutic option for growing patients with mandibular retrognathism. It depends on maintaining the mandible in an advanced position, which produces muscular tension [5]. Functional appliances, either fixed or removable, can transmit these stresses to the skeletal and dental structures, resulting in a combination of skeletal, dentoalveolar, and soft-tissue modifications [6,7]. While some researchers report effective treatment results from mandibular advancement, including length augmentation [8] or condylar growth [9], others dispute these changes [10]. Furthermore, some researchers support the restriction impact on the maxilla, while others found that the change was insignificant [11].

Zhang et al. found that the increase in the mandibular body length following functional therapy with Twin-block was only 2.2 mm after 12 months of active treatment [12], contrary to the study of Lombardo et al., which indicated an increase of around 8.4 mm after the same time of the active treatment [13]. Regarding the maxilla, Namera et al. reported that treatment with the Twin-block appliance limited maxillary growth, accompanied by a slight increase in the SNA angle of approximately 0.42° [14]. In contrast, Baysal et al. observed a reduction in the SNA angle following Twin-block therapy, suggesting that the appliance contributed to correcting the skeletal Class II relationship through maxillary retrusion [15].

Over the last 10 years, four systematic reviews have been published on this topic. These reviews evaluated the skeletal changes following functional therapy with either fixed or removable appliances [16-19]. However, all these reviews may be out of date, as seven years have passed since the last published one. The 2016 systematic review by Pacha et al. [19] compared the effectiveness of fixed versus removable functional appliances. The study provided valuable insights into treatment outcomes; however, it did not consider critical vertical skeletal changes, including alterations in vertical growth patterns, maxillary and mandibular rotation, mandibular height, and total facial height. Furthermore, the absence of a meta-analytic approach limited the ability to synthesize data across studies and draw more generalized conclusions.

Whereas the systematic review of Santamaría-Villegas et al. [16] focused only on the mandibular length changes after functional treatment without any assessment of the other skeletal possible changes. The rest of the reviews can be considered out of date [17,18] given the expected emergence of several randomized controlled trials (RCTs) and controlled clinical trials (CCTs) on this topic. The focused question of this systematic review was as follows: "What are the effects of removable functional appliances (RFAs) and fixed functional appliances (FFAs) on skeletal and dentoalveolar changes in growing patients, compared with untreated controls or alternative appliances, based on evidence from randomized and controlled clinical trials?"

## Review

Material and methods

This systematic review was registered at the initial stage of the study in the PROSPERO database (ID: CRD42025633113) and was conducted in line with the Preferred Reporting Items for Systematic Reviews and Meta-Analyses (PRISMA) guidelines [20].

Eligibility Criteria

The eligibility criteria were established using the Population, Intervention, Comparison, Outcomes, and Study design (PICOS) framework, which also informed the development of the research question and guided the study selection process. The target population consisted of healthy growing individuals diagnosed with skeletal Class II malocclusion. For studies involving removable functional appliances, participants were aged between 9 and 13.5 years, corresponding to the pre-peak to early peak growth stages. For fixed functional appliances, eligible participants were aged between 11 and 14 years, representing the peak-to-early post-peak growth period. No restrictions were applied regarding race.

Comparison groups consisted of either untreated subjects or patients treated with functional appliances different from those used in the experimental group. Only RCTs and CCTs were eligible for inclusion. All studies published up to September 2025 were considered eligible.

The primary outcome measures focused on skeletal and dentoalveolar changes assessed using lateral cephalometric radiographs. Studies were excluded if they were case reports or case series, retrospective in design, animal or in vitro studies, editorials, opinion papers, or technique descriptions. In addition, studies were excluded if they lacked clear sample characterization or if outcomes were assessed using non-cephalometric imaging methods or electromyographic analysis.

Search Strategy

A systematic electronic search was carried out using PubMed®, Web of Science™, Scopus®, Embase®, Google™ Scholar, and the Cochrane Central Register of Controlled Trials (CENTRAL) to retrieve relevant studies published up to September 2025. Additionally, the World Health Organization International Clinical Trials Registry Platform (ICTRP) and ClinicalTrials.gov were searched to detect completed, ongoing, or unpublished clinical trials. No restrictions were applied regarding age, sex, ethnicity, or language. The keywords used in the electronic search are given in Table 1, whereas the details of the search strategy are presented in Table 2.

**Table 1:** Keywords used in the electronic search SNA: the angle between the anterior cranial base and the NA plane; SNB: the angle between the anterior cranial base and the NB plane; ANB: the angle between the NA plane and the NB plane

Aspects of the search strategy	Keywords or related terms
Type of malocclusion	Skeletal Class II, distal occlusion, mandibular retrognathia, increased overjet, excessive overjet
Treatment planning	Functional treatment, growth modification, orthopedic treatment, mandibular advancement, mandibular enlargement
Outcomes	Hard tissue changes, the amount of ANB correction, the amount of SNA change, the amount of SNB change, Dentoalveolar changes, upper incisor retrusion, lower incisor protrusion, the lower facial height change
Intervention	Removable functional appliances, activator, Frankle regulator, Twin-block, Trainer, Bionater, Bite jumping appliance, Hotz, Dynamax, Bimler appliance, bass appliance, fixed functional appliances, AdvanSync, Forsus fatigue-resistant device, Herbst, Jasper jumper

**Table 2:** Electronic search strategy

Database	Search Strategy
Cochrane Central Register of Controlled Trials (CENTRAL)	#1 "class II malocclusion " OR " skeletal class II" OR "distal occlusion" OR "mandibular retrognathia" OR " mandibular retrognathism" #2 "growth modification" OR "functional treatment" OR "jaw relationship correction" OR " mandibular advancement" OR "mandibular enlargement" OR "mandibular protrusion" OR "maxillary restriction" OR" mandibular protrusion appliance" OR " Mandibular anterior repositioning appliance" OR "removable functional appliance" OR" fixed functional appliance" OR "Activator" OR "Frankle regulator" OR "Bionater" OR "Twin-block" OR "Herbst" OR "modified Herbst" OR "Hotz" OR "Trainers " OR " Double plates" OR "Dynamax" OR "Miniblock" OR "Jasper jumper " OR " Forsus nitinol flat spring" OR "Forsus device" OR " Sabbagh spring " OR " Bimler appliance" OR " Bass appliance" OR "Andresen appliance" #3 hard tissue change" OR "mandibular position" OR" dentoalveolar change" OR "mandibular length #4, #1 AND #2 AND #3
Excerpta Medica database (Embase)	#1 "class II malocclusion " OR " skeletal class II " OR "distal occlusion" OR "mandibular retrognathia" OR "mandibular retrognathism" #2 "growth modification" OR "functional treatment" OR "jaw relationship correction" OR " mandibular advancement" OR "mandibular enlargement" OR "mandibular protrusion" OR "maxillary restriction" OR "mandibular protrusion appliance" OR "Mandibular anterior repositioning appliance" OR "removable functional appliance" OR" fixed functional appliance" OR "Activator" OR "Frankle regulator" OR "Bionater" OR "Twin-block" OR "Herbst" OR "modified Herbst" OR "Hotz "OR "Tainers " OR " Double plates" OR "Dynamax" OR "Miniblock" OR "Jasper jumper" OR "Forsus nitinol flat spring" OR "Forsus device" OR "Sabbagh spring" OR " Bimler appliance" OR "Bass appliance" OR "Andresen appliance" #3 hard tissue change" OR " mandibular position" OR" dentoalveolar change" OR "mandibular length" #4, #1 AND #2 AND #3
PubMed	#1 "class II malocclusion " OR " skeletal class II " OR "distal occlusion" OR "mandibular retrognathia" OR " mandibular retrognathism" #2 "growth modification" OR "functional treatment" OR "jaw relationship correction" OR "mandibular advancement" OR "mandibular enlargement" OR " mandibular protrusion" OR "maxillary restriction" OR "mandibular protrusion appliance" OR "Mandibular anterior repositioning appliance" OR "removable functional appliance" OR" fixed functional appliance" OR "Activator" OR "Frankle regulator" OR "Bionater" OR "Twin-block" OR "Herbst" OR "modified Herbst" OR "Hotz" OR "trainers " OR " Double plates" OR "Dynamax" OR "Miniblock “OR"Jasper jumper "OR" Forsus nitinol flat spring" OR "Forsus device" OR "Sabbagh spring " OR "Bimler appliance " OR " Bass appliance" OR "Andresen appliance" #3 " hard tissue change" OR "mandibular position" OR" dentoalveolar change" OR "mandibular length" #4 #1 AND #2 AND #3
Scopus	#1TITLE-ABS-KEY ("class II malocclusion" OR " skeletal class II" OR "distal occlusion" OR "mandibular retrognathia" OR " mandibular retrognathism") #2TITLE-ABS-KEY ("growth modification" OR "functional treatment" OR "jaw relationship correction" OR "mandibular advancement" OR "mandibular enlargement" OR " mandibular protrusion" OR "maxillary restriction" OR "mandibular protrusion appliance" OR "Mandibular anterior repositioning appliance" OR "removable functional appliance" OR" fixed functional appliance" OR "Activator" OR "Frankle regulator" OR "Bionater" OR "Twin-block" OR "Herbst" OR "modified Herbst" OR "Hotz "OR "trainers " OR " Double plates" OR "Dynamax" OR "Miniblock" OR "Jasper jumper" OR " Forsus nitinol flat spring" OR "Forsus device" OR "Sabbagh spring" OR "Bimler appliance" OR "Bass appliance" OR "Andresen appliance") #3TITLE-ABS-KEY (" hard tissue change" OR "mandibular position" OR" dentoalveolar change" OR " mandibular length") #5 #1 AND #2 AND #3
Web of Science	#1 TS= ("class II malocclusion" OR "skeletal class II" OR "distal occlusion" OR "mandibular retrognathia" OR "mandibular retrognathism") #2 TS= ("growth modification" OR "functional treatment" OR "jaw relationship correction" OR "mandibular advancement" OR "mandibular enlargement" OR "mandibular protrusion" OR "maxillary restriction" OR "mandibular protrusion appliance" OR "Mandibular anterior repositioning appliance" OR "removable functional appliance" OR" fixed functional appliance" OR "Activator" OR "Frankle regulator" OR "Bionater" OR "Twin-block" OR "Herbst" OR "modified Herbst" OR "Hotz" OR "trainers " OR " Double plates" OR "Dynamax" OR "Miniblock" OR"Jasper jumper" OR " Forsus nitinol flat spring" OR "Forsus device" OR "Sabbagh spring" OR " Bimler appliance " OR " Bass appliance" OR "Andresen appliance") #3 TS= (" hard tissue change" OR "mandibular position" OR" dentoalveolar change" OR "mandibular length”) #5 #1 AND #2 AND #3
Google Scholar	#1")class II malocclusion " OR " skeletal class II" OR "distal occlusion" OR "mandibular retrognathia" OR" mandibular retrognathism") AND ("growth modification" OR "functional treatment" OR " "jaw relationship correction" OR "mandibular advancement" OR "mandibular enlargement" OR " mandibular protrusion" OR "maxillary restriction" OR" mandibular protrusion appliance" OR "Mandibular anterior repositioning appliance" OR "removable functional appliance" OR" fixed functional appliance" OR "Activator" OR "Frankle regulator" OR "Bionater" OR "Twin-block" OR "Herbst" OR "modified Herbst" OR "Hotz" OR "trainers" OR " Double plates" OR "Dynamax" OR "Miniblock" OR "Jasper jumper" OR "Forsus nitinol flat spring" OR "Forsus device" OR "Sabbagh spring" OR "Bimler appliance "OR" Bass appliance" OR "Andresen appliance) AND ("hard tissue change" OR "mandibular position" OR "dentoalveolar change" OR "mandibular length”)
World Health Organization (WHO) International Clinical Trials Registry Platform (ICTRP) Search Portal	(Functional orthodontic treatment or mandibular advancement or functional appliance or mandibular protrusion appliance) AND (Hard tissue changes OR skeletal and dentoalveolar change)
ClinicalTrials.gov	(Functional orthodontic treatment or mandibular advancement or functional appliance or mandibular protrusion appliance) AND (dentoskeletal change OR skeletal and dentoalveolar change)

Study Selection

DMA and MYH independently assessed the studies and determined whether they met the inclusion criteria for this systematic review. When disagreement arose, a third author, MKA, assisted in its resolution.

Initially, each study was screened based on its title and abstract. When eligibility could not be determined, the full-text article was reviewed. Studies with insufficient information in the title and abstract were also subjected to full-text assessment. Both authors (DMA, MYH) collaboratively contributed to the data extraction process. MKA was asked to resolve disagreements. General information (authors' names, year of publication, and study location), methods (study design, treatment comparison), participants (sample size, age, and gender), intervention (the type of construction bite used), orthodontic aspects (malocclusion characteristics and duration of active treatment), and results were all included in the data summary tables.

Assessment of the Risk of Bias in Included Studies and the Quality of Evidence

The methodological quality of the included studies was independently evaluated by two reviewers (DMA and MYH) using the Cochrane Risk of Bias 2.0 (RoB 2) tool for RCTs [21]. Risk-of-bias domains in randomized trials were classified as low, high, or having some concerns. In cases of disagreement, a third reviewer (MKA) was consulted to reach a consensus. The same reviewers assessed non-randomized studies using the Cochrane Risk of Bias in Nonrandomized Studies of Interventions (ROBINS-I) tool [22]. The certainty of evidence and strength of the conclusions were evaluated using the Grading of Recommendations Assessment, Development, and Evaluation (GRADE) approach [23]. The level of evidence was rated as high, moderate, low, or very low.

Statistical Analysis

Quantitative data synthesis was conducted using Review Manager (RevMan) software (version 5.4; Copenhagen: The Nordic Cochrane Centre, Cochrane Collaboration, London, UK). Both clinical and statistical heterogeneity were assessed across the included studies. Clinical heterogeneity was evaluated by examining differences in treatment protocols, including the duration of active treatment and the characteristics of the comparison groups. Statistical heterogeneity was analyzed using the chi-square (χ²) test, with a significance level set at p < 0.05. In addition, the I² statistic was used to measure the extent of variability attributable to heterogeneity rather than chance, providing an estimate of inconsistency among the included studies.

Results

Study Selection and Inclusion Into the Review

**Figure 1:**
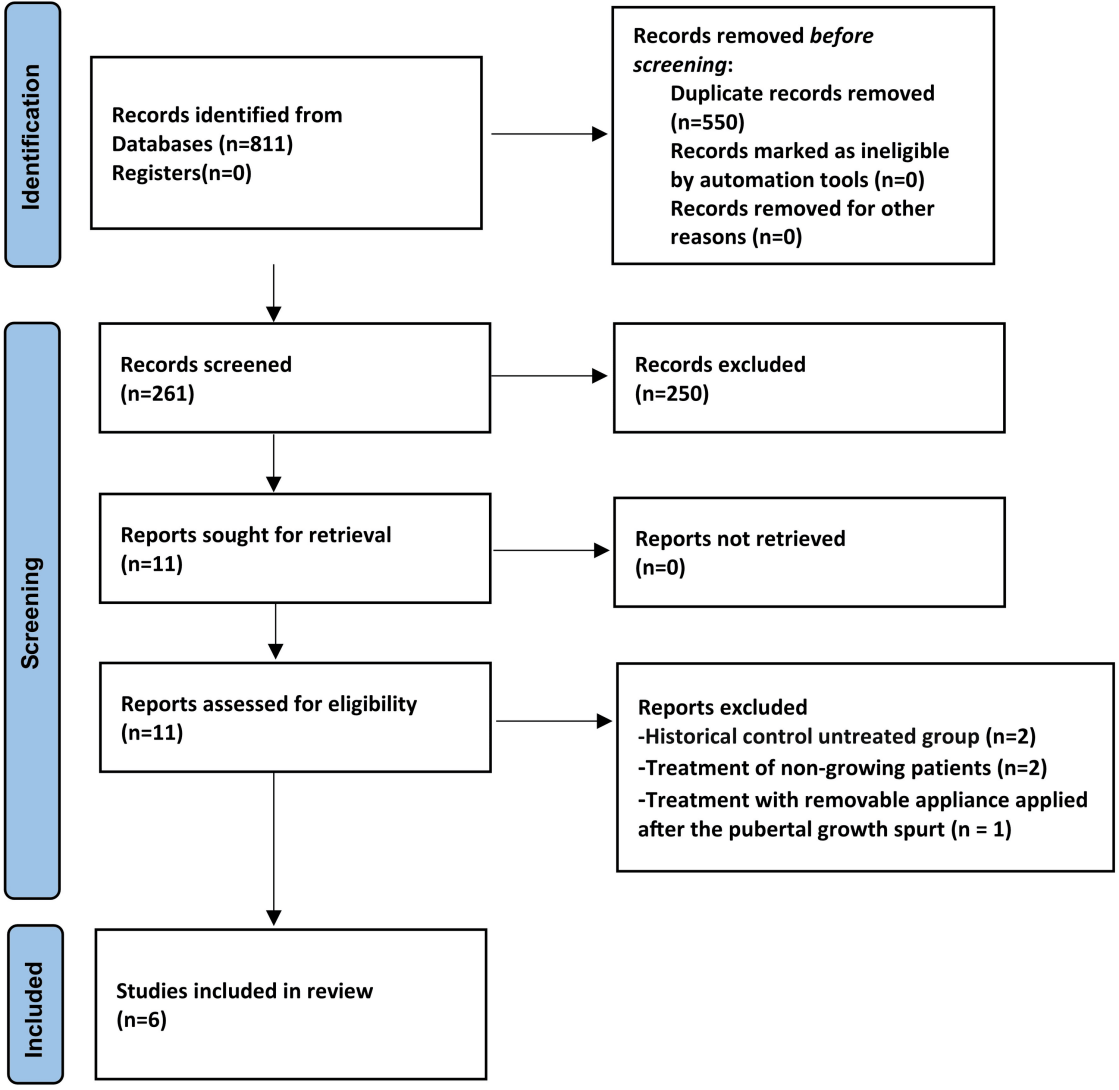
Preferred Reporting Items for Systematic Reviews and Meta-Analyses (PRISMA) flow diagram of the included studies

The PRISMA flow diagram illustrating study identification, screening, eligibility, and inclusion is presented in Figure 1. After removing duplicates, 811 records were retrieved from electronic databases. A total of 261 titles and abstracts were screened, and the full texts of 11 potentially relevant articles were assessed for eligibility. Five studies were excluded as they did not meet the inclusion criteria; the reasons for exclusion are provided in Appendix 1. Ultimately, six studies were included in the systematic review.

Characteristics of the Included Studies

Table 3 illustrates the characteristics of the six included trials. Four RCTs and two CCTs included 276 patients of both genders. The ages of the patients participating in the study were 13.5 years for fixed functional devices and 9-12.5 years for removable ones. Of the six studies, five evaluated removable appliances [24-28], whereas the remaining studies compared fixed appliances with normal growth changes [29].

**Table 3:** Characteristics of the included studies RCT: Randomized clinical trial; CCT: Controlled clinical trials; EXP: Experimental group; ACT: Activator appliance; TB: Twin-block appliance; BJA: Bite-Jumping Appliance; FLMGM: Fixed lingual mandibular growth modificator; ATB: aesthetic Twin-block; CTB: conventional Twin-block; M: male; F: female, NR: Not reported; Bjork's sum: the sum of NSAr, SArGo, and ArGoMe angles; Y-axis: the angle between the anterior cranial base and the Y-axis (measured from Sella to Gnathion) point; MM: the angle between the maxillary plane and the mandibular plane; Jarabak's ratio: the ratio between the posterior facial height (measured from Sella to Gonion) and the anterior facial height (measured from Nasion to Menton)

Study/setting	Study design	Groups of patients	Number of patients (M/F); age in years	Inclusion criteria	Construction bite	Duration of Active Treatment	Outcomes
Quintão et al. [25], Brazil	CCT	TB vs. untreated group	38 (24/14); 9.7 year	Skeletal Class II relationship (ANB > 4 degrees), mandibular retrognathia	Stepwise mandibular advancement	12 months	Incisor inclination, mandibular position, maxillary position
Varlik et al. [24], Turkey	CCT	TB vs. ACT vs. untreated group	50 (25/25); 11.9 ± 0.16 years	Skeletal Class II relationship (ANB > 4 degrees), mandibular retrognathia	Single-step mandibular advancement	ACT: 9 months, TB: 8 months	Incisor inclination, mandibular position, maxillary position
Alali [29], Syria	RCT	FLMGM vs. untreated group	38 (17/21); FLMGM: 13.5 years C: 12.5 years	Skeletal Class II relationship (ANB > 4 degrees), mandibular retrognathia	Single-step mandibular advancement	8 months	Incisor inclination, mandibular position, maxillary position, Jarabak’s ratio, MM angle, overjet
Burhan et al. [27], Syria	RCT	TB vs. BJA	44 (22/22); 10.2-13.5 years	Skeletal Class II relationship (ANB > 4 degrees), mandibular retrognathia	Stepwise mandibular advancement	12 months	Incisor inclination, mandibular position, maxillary position, Bjork sum, Jarabak’s ratio, MM angle
Idris et al. [26], Syria	RCT	ACT vs. Trainer	54 (28/26); 8-12 years	Skeletal Class II relationship (ANB > 4 degrees), mandibular retrognathia	Single-step mandibular advancement	12 months	Incisor inclination, mandibular position, maxillary position, Y-axis, MM angle, mandibular body length, ramus height, maxillary length
Alsilq et al. [28], Syria	RCT	ATB vs. CTB	52 (19/33); 12.33 ± 0.77	Skeletal Class II relationship (ANB > 4 degrees), mandibular retrognathia	Single-step mandibular advancement	NR	Overjet/overbite, incisor inclination, mandibular position, maxillary position, Bjork sum, Jarabak’s ratio, Y-axis, MM angle

The Twin-block appliance was evaluated in four trials, including comparisons with the Bite-jumping appliance (BJA) [27], Activator [24], Aesthetic Twin-block (ATB) [28], and normal growth changes [25]. On the other hand, the Activator was evaluated in a single trial that compared it with the Trainer (T4K®) [26]. Regarding the fixed functional appliance, the fixed lingual mandibular growth modificator (FLMGM) was compared in one study to normal growth [29]. The functional appliances were constructed with the mandible protruding in an edge-to-edge relationship with the incisors [24,26,28,29]. However, mandibular advancement during the construction bite was performed in two steps in the two other trials [25,27].

Cephalometric radiographs were used in all studies to assess hard-tissue changes following therapy. All studies evaluated the angles between the anterior cranial base and the NA plane (SNA), the anterior cranial base and the NB plane (SNB), and the NA and NB planes (ANB). Regarding the vertical dimension, two studies assessed the sum of NSAr, SArGo, and ArGoMe angles (Bjork's sum) [27,28], while three studies assessed the ratio between the posterior facial height (measured from Sella to Gonion) and anterior facial height (measured from Nasion to Menton) (Jarabak's ratio) [27-29]. In addition, four studies assessed the angle between the maxillary plane and the mandibular plane (MM angle) [26-29], and two evaluated the angle between the anterior cranial base and the Y-axis (Sella to Gnathion) [26,28]. Regarding dentoalveolar changes, all studies evaluated changes in incisor inclination.

Risk of Bias of the Included Studies

**Figure 2:**
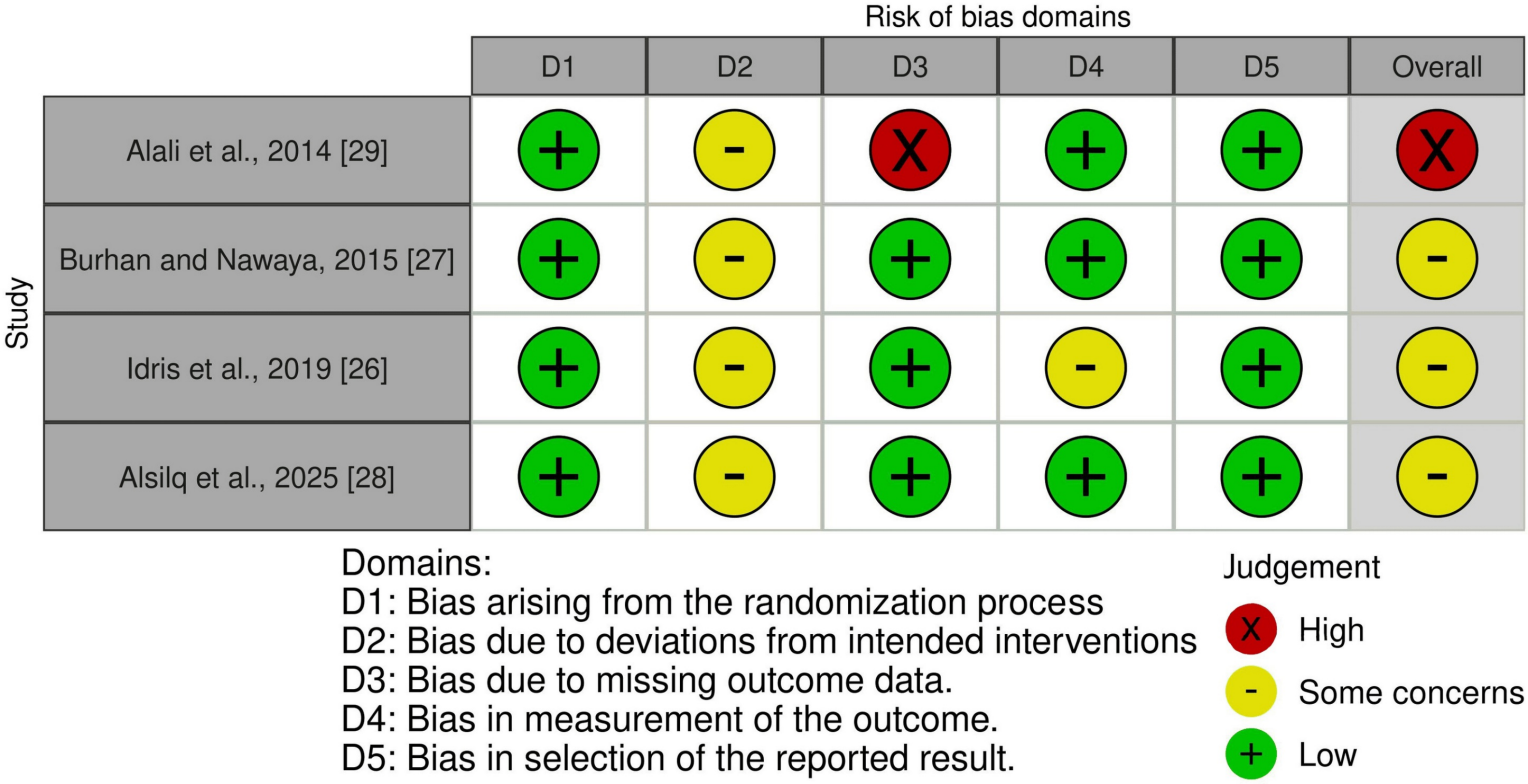
Summary of risk of bias assessment across randomized clinical trials Source: Refs [26-29]

**Figure 3:**
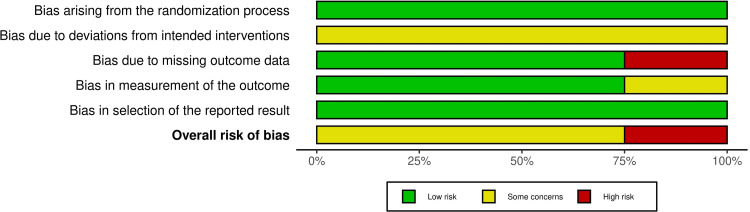
Overall risk of bias scores across different domains in randomized clinical trials

The risk of bias for the four included RCTs is shown in Figure 2, while Figure 3 shows the overall risk of bias across all domains. Further details regarding the evaluation and supporting explanations are shown in Appendix 2. One trial was classified as "high risk of bias" [29], whereas the remaining three RCTs were rated as "some concerns" [26-28].

**Figure 4:**
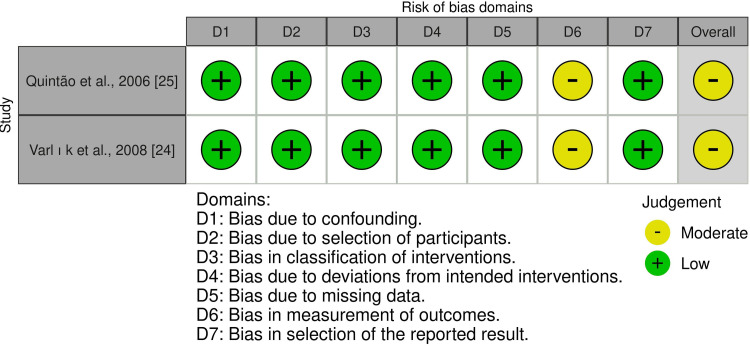
Summary of risk of bias assessment in controlled clinical trials Source: Refs [24,25]

**Figure 5:**
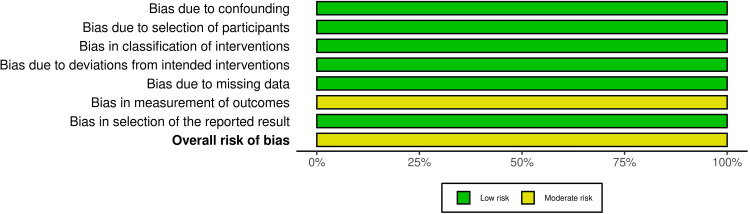
Overall risk of bias scores across different domains in controlled clinical trials

On the other hand, Figure 4 presents an assessment of the risk of bias for the two CCTs, while Figure 5 presents the overall risk of bias for every domain. The two CCTs were rated as having a "moderate risk of bias" [24,25]. Appendix 3 provides more information on the assessment and supporting reasons.

Effects of Interventions

**Figure 6:**
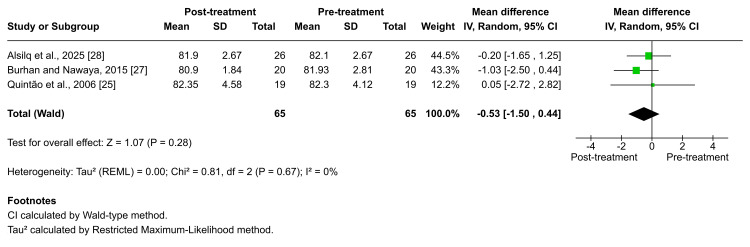
Forest plot of the mean difference in the angle between the anterior cranial base and the NA plane (SNA angle) between baseline and end of active treatment with Twin-block Source: Refs [25,27,28]

Table 4 presents a summary of the main findings. Regarding the SNA angle, five studies evaluated changes in SNA angle following treatment with various functional appliances [25-29]. Among these, Burhan et al., Quintão et al., and Alsilq et al. specifically assessed the Twin-block appliance [25,27,28]. The pooled analysis showed no statistically significant change in the SNA angle at the end of active treatment (MD = -0.53; 95% CI: -1.50 to 0.44; p = 0.28; I² = 0%; Figure 6), with no evidence of heterogeneity. The certainty of evidence for this outcome was rated as moderate. Comparable findings were also observed with other functional devices. Burhan et al., Alsilq et al., and Alali documented no significant post-treatment changes in SNA following the use of the BJA, ATB, and FLMGM appliances [27-29]. In contrast, significant reductions in SNA were reported by Idris et al. following treatment with the Trainer for Kids (T4K®) and the Activator, with mean decreases of ×̅ = -0.56 ± 1.21 (p = 0.021) and ×̅ = -0.59 ± 1.12 (p = 0.011), respectively [26].

**Table 4:** Main results from the included studies TB: Twin-block; C: control untreated group; ACT: Activator; FLMGM: fixed lingual mandibular growth modificator; BJA: Bite-jumping appliance; T4K®: Trainer; ATB: Aesthetic Twin-block; NR: not reported; SNA: the angle between the anterior cranial base and the NA plane; SNB: the angle between the anterior cranial base and the NB plane; ANB: the angle between the NA plane and the NB plane; Bjork's sum: the sum of NSAr, SArGo, and ArGoMe angles; Y-axis: the angle between the anterior cranial base and the Y-axis (Sella to Gnation) point; MM: the angle between the maxillary plane and the mandibular plane; Jarabak's ratio: the ratio between the posterior facial height (measured from Sella to Gonion) and the anterior facial height (measured from Nasion to Menton); A-Y: Horizontal distance from Point A to pterygoid vertical line indicating maxillary position; B-Y: Horizontal distance from Point B to pterygoid vertical line indicating mandibular position Source: Refs [24-29]

Variable	Quintão et al. [25]		Varlik et al. [24]		Alali [29]		Burhan et al. [27]		Idris et al. [26]		Alsliq et al. [28]	
	TB	C	TB	ACT	FLMGM	C	TB	BJA	ACT	T4K®	ATB	TB
SNA	0.05 ± 1.07	0.95 ± 2.37	NR	NR	-0.4 ± 0.7	0.2 ± 0.8	−1.03 ± 0.15	−0.42 ± 0.50	−0.59 ± 1.12	−0.56 ± 1.21	-0.10 ± 1.33	-0.20 ± 1.51
A-Y	NR	NR	1.21 ± 1.91	0.14 ± 3.05	NR	NR	NR	NR	NR	NR	NR	NR
SNB	1.38 ± 1.05	0.92 ± 2.01	NR	NR	1.7 ± 1.1	-0.2 ± 0.7	3.13 ± 1.86	2.88 ± 1.32	1.30 ± 1.11	0.34 ± 1.49	2.72 ± 1.54	1.72 ± 1.14
B-Y	NR	NR	4.37 ± 4.30	4.68 ± 3.57	NR	NR	NR	NR	NR	NR	NR	NR
ANB	–1.33 ± 0.68	0.03 ± 1.20	NR	NR	-2.0 ± 0.9	0.4 ± 0.7	-3.95 ± 0.97	-3.15 ± 1.28	-1.89 ± 1.12	-0.9 ± 1.01	-2.70 ± 0.84	1.92 ± 0.81
Bjork’s sum	NR	NR	NR	NR	NR	NR	-1.12 ± 4.35	3.04 ± 5.69	NR	NR	0.88 ± 3.27	0.08 ± 4.11
Jarabak’s ratio	NR	NR	NR	NR	0.5 ± 1.0	-0.2 ± 1.1	1.26 ± 0.76	-1.78 ± 0.58	NR	NR	0.84 ± 1.44	-0.65 ± 1.37
MM angle	NR	NR	NR	NR	0.2 ± 0.9	0.1 ± 1.3	−0.58 ± 1.60	1.08 ± 2.32	-0.42 ± 2.43	0.09 ± 2.08	0.10 ± 2.62	-0.32 ± 2.37
Y-axis	NR	NR	NR	NR	NR	NR	NR	NR	-0.15 ± 1.78	0.23 ± 1.23	-0.06 ± 1.63	-0.20 ± 1.42
Upper incisor inclination	-7.98 ± 4.79	-0.05 ± 3.37	-2.16 ± 0.91	-2.82 ± 0.09	-3.1 ± 2.8	0.9 ± 2.7	-4.12 ± 1.83	-3.78 ± 1.07	-3.17 ± 7.32	-2.37 ± 1.72	-2.00 ± 2.02	-4.18 ± 3.34
Lower incisor inclination	3.13 ± 3.04	0.71 ± 3.15	1.86 ± 0.95	2.71 ± 1.38	-4.1 ± 4.7	0.4 ± 1.4	3.63 ± 1.62	3.25 ± 2.38	0.47 ± 1.57	1.07 ± 3.59	1.34 ± 2.08	3.88 ± 5.86

**Figure 7:**
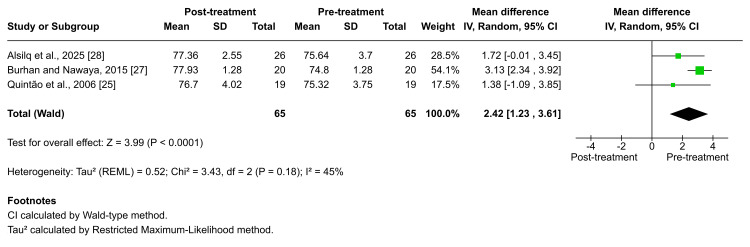
Forest plot of the mean difference in the angle between the anterior cranial base and the NB plane (SNB angle) between baseline and end of active treatment with Twin-block Source: Refs [25,27,28]

Concerning the SNB angle, five studies evaluated changes in SNB angle following functional appliance therapy [25-29]. The studies by Burhan et al., Quintão et al., and Alsilq et al., all investigating the Twin-block appliance, were included in the pooled analysis, which demonstrated a statistically significant increase in SNB; however, the heterogeneity was moderate (MD = 2.42; 95% CI: 1.23-3.61; p < 0.0001; I² = 45%; Figure 7). The certainty of evidence for this outcome was rated as low [25,27,28]. Additional significant increases in SNB were reported across other appliances. Burhan et al., Alsilq et al., Idris et al., and Alali documented notable mandibular advancement following treatment with BJA, ATB, Activator, and FLMGM, respectively (×̅ = 2.88 ± 1.32, p = 0.005; ×̅ = 2.72 ± 1.54, p = 0.000; ×̅ = 1.30 ± 1.11, p < 0.01; ×̅ = 1.7 ± 1.1, p < 0.01, respectively) [26-29]. In contrast, Idris et al. reported that therapy with the T4K® did not result in a statistically significant change in the SNB angle (p = 0.081), suggesting limited mandibular positional effects of this appliance [26].

**Figure 8:**
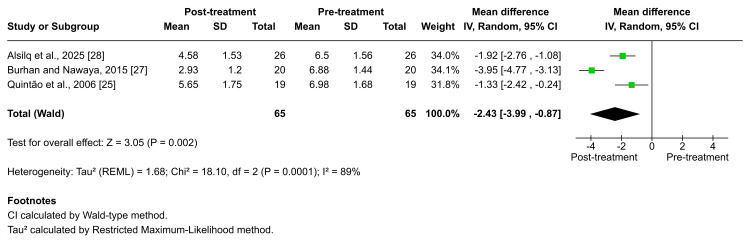
Forest plot of the mean difference in the angle between the NA plane and the NB plane (ANB angle) between baseline and end of active treatment with Twin-block Source: Refs [25,27,28]

When assessing changes in the ANB angle, all five included studies reported a reduction following functional treatment with both fixed and removable appliances. Burhan et al., Quintão et al., and Alsilq et al. evaluated the effects of Twin-block therapy, and the pooled analysis demonstrated a statistically significant decrease in ANB angle after treatment (MD = -2.43; 95% CI: -3.99 to -0.87; p = 0.002; I² = 89%; Figure 8). Heterogeneity across these studies was high, and the certainty of evidence for this outcome was graded as low [25,27,28]. Significant reductions in the ANB angle were also reported following treatment with other removable appliances, including the T4K®, Activator, BJA, and ATB as documented by Idris et al., Burhan et al., and Alsilq et al., respectively (×̅ = -0.9 ± 1.01, p = 0.012; ×̅ =-1.89 ± 1.12, p < 0.001; ×̅ = -3.15 ± 1.28, p = 0.004; ×̅ = -2.70 ± 0.84, p = 0.000) [26-28].

Regarding fixed functional appliances, Alali observed statistically significant reductions in the ANB angle following treatment with FLMGM (×̅ = -2.0 ± 0.9, p < 0.001) [29]. Despite these favorable skeletal changes with all appliances, patients generally completed treatment with a residual mild Class II skeletal pattern.

**Figure 9:**
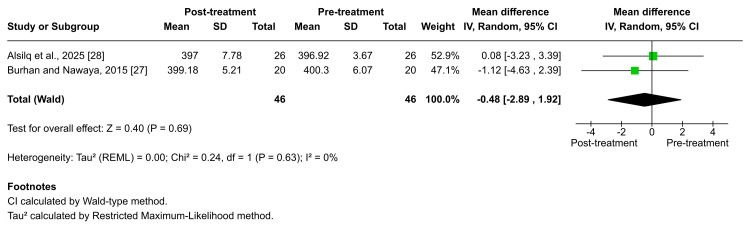
Forest plot of the mean difference in the sum of NSAr, SArGo, and ArGoMe angles (Bjork's sum) between baseline and end of active treatment with Twin-block Source: Refs [27,28]

Bjork's sum and Y-axis: According to Burhan et al. and Alsilq et al., Bjork’s sum showed no significant change following Twin-block functional therapy [27,28]. The pooled estimate similarly showed no meaningful effect (MD = -0.48; 95% CI: -2.89 to 1.92; p = 0.69; I² = 0%; Figure 9), indicating no heterogeneity. The certainty of evidence for this outcome was rated as moderate [27,28]. Similarly, no significant post-treatment changes in Bjork's sum were reported after therapy with the ATB or the BJA, as documented by Burhan et al. and Alsilq et al. (p = 0.266 and p = 0.095, respectively) [27,28]. Two studies assessed alterations in the Y-axis following treatment with removable functional appliances [26,28]. Both studies consistently reported no significant differences after therapy with the Activator, T4K®, ATB, or Twin-block (p = 0.645, p = 0.316, p = 0.855, and p = 0.494, respectively).

**Figure 10:**
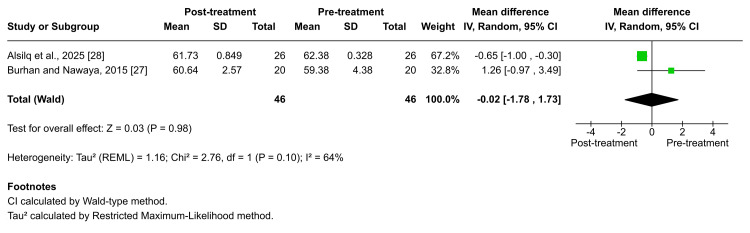
Forest plot of the mean difference in posterior facial height and anterior facial height (Jarabak's ratio) between baseline and end of active treatment with Twin-block Source: Refs [27,28]

Jarabak's ratio: According to Burhan et al. and Alsilq et al., Jarabak's ratio did not exhibit a significant change following Twin-block functional therapy [27,28]. The pooled estimate confirmed the absence of a meaningful alteration (MD = -0.02; 95% CI: -1.78 to 1.73; p = 0.98; I² = 64%; Figure 10), reflecting moderate heterogeneity. The certainty of evidence for this outcome was rated as low [27,28]. Consistent findings were reported by Alali, who observed no significant change in Jarabak's ratio after treatment with the FLMGM appliance (×̅ = 0.5 ± 1.0) [29]. In contrast, Alsilq et al. [28] observed a statistically significant increase in Jarabak's ratio following treatment with the ATB (×̅ = 0.84 ± 1.44, p = 0.007), reflecting a relative decrease in lower anterior facial height. Furthermore, Burhan et al. recorded a significant reduction in Jarabak's ratio after BJA therapy (×̅ = -1.78 ± 0.58; p = 0.002), indicating an increase in vertical facial height [27].

**Figure 11:**
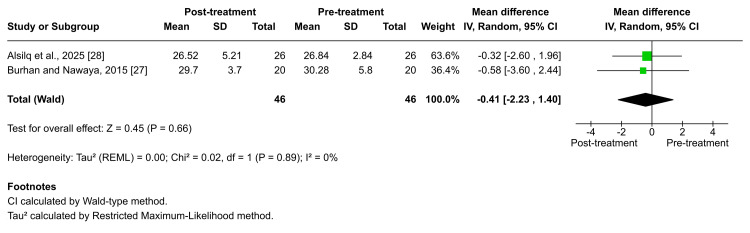
Forest plot of the mean difference of the angle between the maxillary plane and the mandibular plane (MM angle) between baseline and end of active treatment with Twin-block Source: Refs [27,28]

The MM angle: According to Burhan et al. and Alsilq et al., the MM angle did not exhibit a significant change following Twin-block functional therapy [27,28]. The pooled estimate likewise confirmed the absence of a meaningful alteration (MD = -0.41; 95% CI: -2.23 to 1.40; p = 0.66; I² = 0%; Figure 11), indicating no heterogeneity. The certainty of evidence for this outcome was rated as moderate. Similarly, Idris et al., Alsilq et al., and Alali [26,28,29] reported no significant changes in the MM angle following treatment with the T4K®, Activator, ATB, and FLMGM appliances (p = 0.599, p = 0.353, p = 0.855, respectively), further supporting the stability of this vertical skeletal parameter across different functional therapy modalities. In contrast, Burhan et al. demonstrated a significant increase in the MM angle following treatment with the BJA (x̅ = 1.08 ± 2.32, p = 0.002) [27].

**Figure 12:**
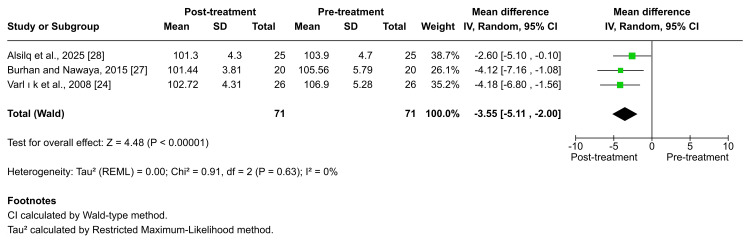
Forest plot of the mean difference in the angle between the anterior cranial base and the upper incisor axis (UI-SN angle) between baseline and end of active treatment with Twin-block Source: Refs [24,27,28]

Upper incisors' inclination: According to Varlik et al., Burhan et al., and Alsilq et al. [24,27,28], treatment with the Twin-block appliance resulted in a statistically significant retrusion of the upper incisors. The pooled analysis likewise confirmed a significant posterior tipping (MD = -3.55; 95% CI: -5.11 to -2.00; p < 0.00001; I² = 0%; Figure 12), indicating no heterogeneity. The certainty of evidence for this outcome was rated as moderate. Significant upper-incisor retrusion was also reported following treatment with other functional appliances, including the BJA, T4K®, Activator, Twin-block, and FLMGM, as documented by Burhan et al., Idris et al., Varlik et al., Quintão et al., and Alali et al. [24-27,29] (x̅ = -3.78 ± 1.07, p = 0.002; x̅ = -2.37 ± 1.72, p = 0.025; x̅ = -3.17 ± 7.32, p = 0.021; x̅ = -2.82 ± 0.09, p ≤ 0.001; x̅ = -7.98 ± 4.79, p < 0.001; x̅ = -3.10 ± 2.80, p < 0.001, respectively). However, in contrast to these findings, Alsilq et al. reported no significant change in upper-incisor inclination following treatment with the ATB appliance (p = 0.716) [28].

**Figure 13:**
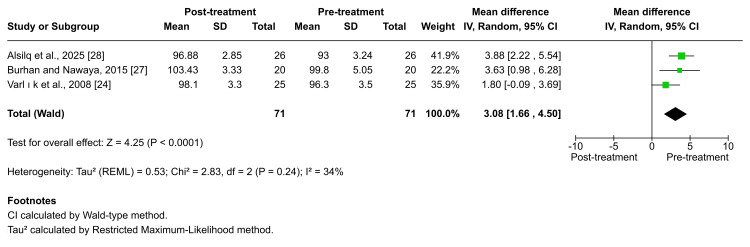
Forest plot of the mean difference in the angle between the mandibular plane and the lower incisor axis (LI-MP angle), between baseline and end of active treatment with Twin-block Source: Refs [24,27,28]

Lower incisors' inclination: A significant proclination of the lower incisors following Twin-block therapy was consistently reported by Burhan et al., Varlik et al., and Alsilq et al. [24,27,28]. The pooled meta-analysis similarly demonstrated a statistically significant increase in lower-incisor inclination (MD = 3.08; 95% CI: 1.66-4.50; p < 0.0001; I² = 34%; Figure 13), indicating moderate heterogeneity. The certainty of evidence for this outcome was rated as low.

Comparable findings were observed with other functional appliances. Significant proclination was reported after treatment with the BJA, Twin-block, ATB, and the Activator, as documented by Burhan et al., Quintão et al., Alsilq et al., and Varlik et al., with mean increases of (x̅ = 3.25 ± 2.38, p < 0.007; x̅ = 3.13 ± 3.04, p < 0.05; x̅ = 1.34 ± 2.08, p = 0.004, x̅ = 2.71 ± 1.38, p ≤ 0.001, respectively) [24,25,27,28]. In contrast, Alali reported a significant retrusion of the lower incisors following treatment with the FLMGM appliance (x̅ = -4.10 ± 4.70; p < 0.01), suggesting an appliance-specific effect on lower-incisor inclination [29]. Meanwhile, Idris et al. found no significant post-treatment changes in the position of the lower incisors following the Activator and T4K® (p = 0.573 and p = 0.132, respectively) [26]. Quality of the evidence regarding skeletal changes: Based on the GRADE approach, the quality of evidence for skeletal and dental measurements ranged from moderate to low. Further details can be found in Table 5.

**Table 5:** Summary of the findings from the included studies according to the Grading of Recommendations Assessment, Development and Evaluation (GRADE) approach High quality: Further research is very unlikely to change our confidence in the estimate of effect. Moderate quality: Further research is likely to have an important impact on our confidence in the estimate of effect and may change the estimate. Low quality: Further research is very likely to have an important impact on our confidence in the estimate of effect and is likely to change the estimate. Very low quality: we are very uncertain about the estimate SNA: the angle between the anterior cranial base and the NA plane; SNB: the angle between the anterior cranial base and the NB plane; ANB: the angle between the NA plane and the NB plane; Bjork's sum: the sum of NSAr, SArGo, and ArGoMe angles; Y-axis: the angle between the anterior cranial base and the Y-axis (Sella to Gnathion); MM: the angle between the maxillary plane and the mandibular plane; Jarabak's ratio: the ratio between the posterior facial height (measured from Sella to Gonion) and the anterior facial height (measured from Nasion to Menton). a, Downgraded due to risk of bias. b, Downgraded due to small sample size. c Downgraded due to moderate heterogeneity between studies.

Quality assessment criteria						Summary of findings				Comments
Outcomes	Risk of bias	Inconsistency	Indirectness	Imprecision	Other considerations	No. of patients (trials)	Effects		Certainty	
							Absolute (95% CI)	Relative (95% CI)		
Skeletal change										
SNA										
SNA, change after treatment with Twin-block	Serious	Not serious	Not serious	Not serious	None	130 (3)	-	MD=-0.53; 95%; CI: -1.50, 0.44	Moderate ⊕⊕⊕⊖^a^	The evidence suggests that functional treatment using the Twin-block probably has no effect on the SNA angle.
SNB										
SNB, change after treatment with Twin-block	Serious	Serious	Not serious	Not serious	None	130 (3)	-	MD=2.42; 95%; CI: 1.23, 3.61	Low ⊕⊕⊖⊖^a,c^	The evidence suggests that functional treatment using Twin-block increased the SNB angle, but the evidence is uncertain.
ANB										
ANB, change after treatment with Twin-block	Serious	Serious	Not serious	Not serious	None	130 (3)	-	MD=-2.43; 95% CI: -3.99, -0.87	Low ⊕⊕⊖⊖^a,c^	The evidence suggests that functional treatment using Twin-block decreased the ANB angle, but the evidence is uncertain.
Bjork's sum										
Bjork's sum, change after treatment with Twin-block	Serious	Not serious	Not serious	Not serious	None	92 (2)	-	MD=-0.48; 95% CI: -2.89, 1.92	Moderate ⊕⊕⊕⊖	Functional treatment probably did not change Björk’s sum.
Jarabak's ratio										
Jarabak's ratio, change after treatment with Twin-block	Serious	Serious	Not serious	Not serious	None	92 (2)	-	MD=-0.02; 95% CI: -1.78, 1.73	Low ⊕⊕⊖⊖^a,^^c^	Functional treatment with Twin-block did not change the Jarabak's ratio, but the evidence is uncertain.
The MM angle										
MM angle, change after treatment with Twin-block	Serious	Not serious	Not serious	Not serious	None	92 (2)	-	MD=-0.41; 95% CI: -2.23, 1.40	Moderate ⊕⊕⊕⊖^a^	Functional treatment with Twin-block probably did not change the MM angle.
Dentoalveolar change										
Upper incisors' inclination										
Upper incisors' inclination, change after functional treatment with Twin-block	Serious	Not serious	Not serious	Not serious	None	142 (3)	-	MD=-3.55; 95%; CI: -5.11, -2.00	Moderate ⊕⊕⊕⊖^a^	The evidence suggests that functional treatment with Twin-block significantly retracted the upper incisors.
Lower incisors' inclination										
Lower incisors' inclination, change after treatment with Twin-block	Serious	Serious	Not serious	Not serious	None	142 (3)	-	MD=3.08; 95%; CI: 1.66, 4.50	Low ⊕⊕⊖⊖^a,^^c^	The evidence suggests that functional treatment with Twin-block did not significantly protrude the lower incisors, but the evidence is uncertain.

Discussion

This systematic review assessed the effects of fixed and removable functional appliances on skeletal and dentoalveolar structures. The analysis included data from 276 patients across four RCTs and two CCTs, all evaluating cephalometric changes following Class II correction using RFAs and FFAs. It is important to note that the meta-analysis was limited to only three studies focusing on removable functional appliances, due to the limited availability of comparable quantitative data.

Risk of Bias Across Studies and Additional Analyses

Regarding risk of bias in the included studies, three RCTs were rated as "some concerns" [26-28], and another was rated as "high risk of bias" [29]. Missing outcome data were identified as a source of bias in one trial [29]. Bias due to deviations from the intended interventions was observed in all studies; nonetheless, because blinding the specialist is impossible, this may have influenced the process. Furthermore, bias in outcome measurement was found in one trial [26]. Regarding the two CCTs, they were defined as "moderate risk of bias" [24,25]. Bias in measuring the outcome process was identified as a potential source of bias, as assessors were most likely aware of whether they were receiving therapy, which may have biased the assessment.

Skeletal Changes

The meta-analysis results showed that functional therapy using Twin-block improved the sagittal discrepancy at the end of active treatment, with a combination of maxillary restraint and increased mandibular length, accompanied by an increasing SNB angle and without a change in SNA [25,27,28]. However, the skeletal relationship was categorized as mild Class II (according to the ANB angle) at the end of active treatment.

This improvement was also observed with other appliances, such as the Activator and BJA [26,27]. Although the ATB resulted in greater statistical improvement [28], the differences between the appliances in the skeletal changes achieved were not clinically significant.

On the other hand, this improvement was not observed at the end of active treatment with the Trainer appliance [26]. Unlike conventional removable functional appliances, which are individually fabricated in acrylic and activated according to a construction bite registration, allowing precise anterior mandibular repositioning and providing rigid support to hold the mandible in the advanced position, the Trainer is a prefabricated device made of elastic polyurethane. Its high flexibility makes it difficult for children to maintain the mandible consistently in a forward position during wear. Traditional functional appliances are designed based on specific patient records (construction bite, stage of growth, skeletal discrepancy) and thus act directly on skeletal and dentoalveolar structures by guiding mandibular growth. In contrast, non-traditional prefabricated appliances, such as the Trainer, are primarily intended as myofunctional appliances that target muscular dysfunction, habits, and soft-tissue balance, with only indirect, less predictable skeletal effects. This fundamental difference in design and mechanism of action may explain why the Trainer demonstrates fewer skeletal changes than conventional functional appliances [30]. Therefore, it is important to emphasize this distinction in both clinical use and in future research, where prefabricated appliances should not be considered equivalent to traditional functional appliances [30].

Regarding fixed functional appliances, FLMGM reduced the sagittal discrepancy. Even so, the skeletal relationship was categorized as mild Class II at the end of active treatment according to the ANB angle (mean: -2.0°), and there was no difference compared with Activator and Twin-block, respectively [29].

Vertically, Activator, Twin-block, ATB, and Trainer (T4K®) therapy produced no clinically or statistically significant change in vertical facial height (according to the MM angle, Bjork’s sum, and the Y-axis, Jarabak's ratio) [26-28]. This is explained by the acrylic bite blocks that cover the posterior teeth, restrict their extrusion, and prevent vertical growth. In contrast, the vertical face height increased significantly during BJA functional therapy. These alterations were produced by an acrylic bite block that covered only the anterior teeth [27]. Considering that the bite block is left untrimmed during this phase, and all studies ended the evaluation at the end of active treatment. Regarding fixed functional appliances, vertical facial height did not change significantly after active treatment with FLMGM, which may explain the intrusive effect in the upper posterior region (as indicated by the MM angle and Jarabak's ratio) [29].

Upper and Lower Incisors' Changes

According to the meta-analysis, treatment with the Twin-block appliance resulted in a significant retroclination of the upper incisors. Similarly, functional treatment using the Activator, BJA, and T4K® also produced significant retroclination, with mean changes of -3.17°, -3.78°, and -2.37°, respectively. These changes were assessed using the UI-SN angle (the angle between the upper incisor axis and the anterior cranial base) and the UI-NA angle (the angle between the upper incisor axis and the NA plane) [24-28]. As for fixed appliances, the upper incisors had the same result with the FLMGM appliance, with a mean of -3.1° [29]. This finding can be evaluated as a posterior reaction of the upper incisors due to the mandibular anterior advancement. However, the ATB caused a smaller reduction in upper incisor inclination, with the buccal and palatal surfaces completely covered, thereby limiting the effect of this tilting and resulting in insignificant retraction [28].

Regarding the lower incisors, the meta-analysis showed that lower incisor inclination increased significantly following Twin-block therapy [24,27,28]. This change may be explained by mesial forces acting on the mandible during functional advancement. Treatment with the Activator also resulted in a greater degree of lower incisor proclination, with a mean change of 2.8° according to the LI-MP angle (the angle between the mandibular plane and the lower incisor axis). This effect was more pronounced when the appliance was designed with a labial bow configuration [24]. Another design, an acrylic ledge, caused non-significant changes in inclination [27]. Similarly, ATB caused a non-significant modification in inclination, which can be explained by the complete coverage of the buccal surface cervically of the lower incisors by vacuum-formed hard plates (VFPs) and their rigidity, which limits the effect of the mesial forces resulting from the appliance and reinforced anchorage, providing greater stability in the sagittal dimension [28].

Based on the findings of lower incisor protrusion, we recommend incorporating an acrylic ledge that covers approximately one-third of the buccal surfaces of the lower incisors into removable functional appliances to help prevent excessive labial tipping and improve treatment control.

Treatment with fixed FLMGM appliances resulted in a marked retroclination of the mandibular incisors. This effect may be explained by the absence of mesially directed forces on the lower dentition, as the acrylic inclined plane does not engage the mandibular anterior teeth. In addition, the appliance appears to alter the balance between lingual and labial muscular pressures. The vertical advancement loops reduce tongue pressure on the lower incisors by acting as a barrier, whereas the continuous pressure exerted by the lips promotes lingual tipping of the incisor crowns [29].

We would like to note that this review is largely confirmatory, as the findings align with existing literature. This aspect reinforces the consistency of the evidence regarding the short-term effects of fixed and removable functional appliances and can provide further confidence in clinical decision-making.

Clinical Implications

Based on this review, clinicians should carefully select the type of functional appliance and consider the patient's growth stage when planning Class II treatment. Removable appliances, such as the Activator or Twin-block, are most effective during the peak of the pubertal growth spurt for sagittal corrections, while fixed functional appliances are more suitable at later stages for dentoalveolar adjustments. Patient's vertical facial pattern should also be considered: choose appliances that promote vertical development, such as the BJA, or those that maintain vertical height, such as the Twin-block and Activator, according to the treatment goal. Appliance design, including acrylic ledges or bite registration, can further influence incisor position and vertical facial dimension.

Limitations of the Current Review

The first limitation of this review was the limited number of high-quality RCTs and CCTs; only six studies were included. Another limitation is the short follow-up period, which ended only after active treatment. In addition, there was variability in the amount of mandibular advancement between single-step bite jumping and the stepwise approach. Moreover, the meta-analysis results have been restricted to changes produced by removable functional appliances and are based on only three studies, further limiting the generalizability of the findings. Future studies should focus on addressing these gaps to ensure highly reliable and credible results.

## Conclusions

Based on this review, the following conclusions can be drawn for the short-term effectiveness of FFAs and RFAs. Both types contributed to improving Class II malocclusion with a combination of maxillary restraint and increased mandibular length in addition to the dentoalveolar changes, except for the T4K®; the evidence ranged from moderate to low, largely due to the serious risk of bias in the included studies. Vertically, except for BJA, no clinically notable changes were observed in the short term, with a level of certainty ranging from moderate to low due to the serious risk of bias and heterogeneity within the included studies.

However, more randomized controlled clinical studies comparing fixed and removable appliances, with a follow-up period extending beyond the active phase and establishing correct posterior occlusion, are required for more reliable conclusions.

## Appendices

Appendix 1

**Table 6:** Excluded studies and the reasons beyond exclusion CMVI: Cervical Vertebral Maturation Index

Authors, Year	Study	Reason for exclusion
Gunay et al., 2011	Gunay E.A. Arun T. and Nalbantgil D (2011). Evaluation of the immediate dentofacial changes in late adolescent patients treated with the Forsus(™) FRD. European Journal of Dentistry, 5, 423–432	The treatment was provided to non-growing patients with a Force fatigue-resistant appliance with a cervical skeletal maturity of CMVI5-CMVI6.
Oztoprak et al., 2012	A cephalometric comparative study of class II correction with Sabbagh Universal Spring SUS and Forsus FRD appliances	The treatment was provided to non-growing patients with a Force fatigue-resistant appliance with a cervical skeletal maturity of CMVI5-CMVI6.
Bilgic et al., 2015	Comparison of Forsus FRD EZ and Andresen activator in the treatment of class II, division 1 malocclusions	Patients were treated with the activator appliance after the pubertal growth spurt.
Khoja et al., 2015	Cephalometric evaluation of the effects of the Twin Block appliance in subjects with Class II, Division 1 malocclusion amongst different cervical vertebral maturation stages	The experimental group was compared with a historical control group of untreated patients.
Lombardo et al., 2022	Dentoskeletal effects of clear aligner vs twin block - a short-term study of functional appliances	The experimental groups were compared with a historical control group of untreated patients.

Appendix 2

**Table 7:** Details of the risk of bias assessment of randomized controlled trials

Study	Bias arising from the randomization process	Bias due to deviations from intended interventions		Bias due to missing outcome data	Bias in the measurement of the outcome	Bias in the selection of the reported result	Overall bais
		Effect of assignment to intervention	Effect of adhering to the intervention				
	D1	D2		D3	D4	D5	
Alali, 2014 [29]	Low risk: All subjects were randomized by the author at the beginning of the study to either the treatment or control group.	Some concerns: Blinding cannot be performed. There is ‘No information’ on whether any deviations arose because of the trial context.	Some concerns: Blinding cannot be performed. And ‘No information on whether the important non-protocol interventions were balanced across intervention groups	High risk 5 patients “five patients were lost to follow-up due to different reasons” 11.6% dropped out.	Low risk: blinding of assessment was performed. The method of measuring the outcome is appropriate	Low risk: The trial was not registered in any major database of clinical trials. However, the reported outcomes in the result section seemed to correspond with the pre-defined outcomes aforesaid in the method section.	High risk
Burhan et al., 2015 [27]	Low risk: “Randomization was performed by one of the academic staff at the Orthodontic Department who was not involved directly in this research project using a computer-generated random sequence of numbers.”	Some concerns: Blinding cannot be performed. There is ‘No information’ on whether any deviations arose because of the trial context.	Some concerns: Blinding cannot be performed. And ‘No information on whether the important non-protocol interventions were balanced across intervention groups	Low risk: 4 patients (2 patients in the Twin-block group and 2 in the Bite-Jumping appliance group)	Low risk: blinding of assessment was performed. The method of measuring the outcome is appropriate.	Low risk: No information about the registration protocol was mentioned. However, the reported outcomes in the results section seemed to correspond with the pre-defined outcomes aforesaid in the method section.	Some concerns
Idris et al., 2019 [26]	Low risk: “Randomization was performed by one of the academic staff at the Orthodontic Department who was not involved directly in this research project using a computer-generated random sequence of numbers.”	Some concerns: Blinding cannot be performed. There is ‘No information’ on whether any deviations arose because of the trial context.	Some concerns: Blinding cannot be performed. And ‘No information on whether the important non-protocol interventions were balanced across intervention groups	Low risk: 6 patients (2 patients in the Activator group and 4 in the Trainer group). “Six patients were lost to follow-up due to different reasons.” Nearly all the outcome data is available 90%	Some concerns: “No blinding was applied in this trial.” The method of measuring the outcome is appropriate.	Low risk: The trial was not registered in any major database of clinical trials. However, the reported outcomes in the results section seemed to correspond with the pre-defined outcomes aforesaid in the method section.	Some concerns
Alsilq et al., 2025 [28]	Low risk: A computer-generated randomization list was used to randomly divide the patients into two equal groups via Minitab® Version 19.1 (Minitab Inc., Pennsylvania, USA), which was created by one of the academic staff (not involved in this research) at the Department of Orthodontics.	Some concerns: Blinding cannot be performed. There is ‘No information’ on whether any deviations arose because of the trial context.	Some concerns: Blinding cannot be performed. And ‘No information on whether the important non-protocol interventions were balanced across intervention groups	Low risk: No dropped out	Low risk: blinding of the assessment was performed. The method of measuring the outcome is appropriate	Low risk: The trial was prospectively registered in an international clinical trial registry. The presence of a preregistered protocol reduces the likelihood of selective outcome reporting, and no discrepancies were identified between the reported outcomes and the registered protocol.	Some concerns

Appendix 3

**Table 8:** Details of the risk of bias assessment of the controlled clinical trials Source: Refs [24,25]

Studies								
Quintão et al., 2006 [25]	Low risk: No confounding expected	Low risk: all the participants start of follow-up and the start of the intervention concided	Low risk: Intervention is well defined	Low risk: There were no deviations from the intended intervention beyond what would be expected in usual practice	Low risk: Data were available for all participants	Moderate risk: No information about the outcome assessors' blinding	Low risk: The study reported measurements and analysis of selected variables.	Moderate risk of bias
Varlik et al., 2008 [24]	Low risk: No confounding expected	Low risk: all the participants start of follow-up and the start of intervention concided	Low risk: Intervention is well defined	Low risk: There were no deviations from the intended intervention beyond what would be expected in usual practice	Low risk: Data were available for all participants	Moderate risk: No information about the outcome assessors' blinding	Low risk: The measurements and analysis of selected variables were reported in the study	Moderate risk of bias
